# Targeting FAK to Potentiate Immune Checkpoint Therapy in Solid Tumors

**DOI:** 10.33696/cancerimmunol.7.109

**Published:** 2025

**Authors:** Karly A. Stanley, Sheri L. Holmen

**Affiliations:** 1 Huntsman Cancer Institute, University of Utah Health Sciences Center, Salt Lake City, Utah 84112, USA; 2 Department of Oncological Sciences, University of Utah Health Sciences Center, Salt Lake City, Utah 84112, USA; 3 Department of Surgery, University of Utah Health Sciences Center, Salt Lake City, Utah 84112, USA

**Keywords:** Angiogenesis, Cancer immunotherapy, Combination therapies, Fibrosis, Focal adhesion kinase, Immune checkpoint inhibitors, Tumor microenvironment

## Abstract

The advent of immune checkpoint inhibition revolutionized cancer care, yet many people will fail to respond due to innate or acquired resistance. Combination therapies with immune checkpoint inhibitors are being explored to enhance their efficacy and improve patient outcomes. Focal adhesion kinase (FAK), known for its roles in cell adhesion and migration, has emerged as a potential therapeutic target due to the identification of its additional functions in cancer progression, including its ability to establish a pro-tumorigenic, immunosuppressive microenvironment. FAK has the ability to create physical barriers for immune cell infiltration and drug delivery through its regulation of blood vessel formation and extracellular matrix in the tumor stroma. Additionally, FAK has been reported to function both within tumor and immune cells to inhibit immune cell recruitment, stimulation, and function. Taken together, FAK functions within cancer to dampen immune surveillance and promote immune escape. As a result, there is mounting interest in the use of FAK inhibitors in combination with immune checkpoint inhibition for the treatment of solid tumors, and this strategy is actively being explored in the pre-clinical and clinical setting. This article reviews the ways in which FAK alters the tumor microenvironment and the cells within it in order to assess the clinical potential of co-targeting FAK and immune checkpoints.

## Introduction

The introduction of immune checkpoint inhibition has revolutionized cancer treatment [1,2]. Immune checkpoint inhibition works to eliminate cancer through the stimulation of a patient’s own immune system, allowing immune cells to effectively recognize and eliminate cancer cells. This landmark achievement was propelled by the identification of cytotoxic T-lymphocyte-associated protein 4 (CTLA-4), an inhibitory protein receptor expressed by T cells. The dampening effects of CTLA-4 on T cell function are caused predominately through competitive binding of its ligands with the co-stimulatory molecule CD28. When CTLA-4 outcompetes CD28 for ligand binding, it blocks T cell activation. In effector T cells, CTLA-4 expression on the cell surface is induced by T cell receptor (TCR) signaling. In regulatory T cells (Tregs), CTLA-4 is constitutively expressed and is thought to suppress T cell effector function through downregulation of B7 ligands on antigen presenting cells (APCs). The suppresive effects of CLTA-4 on T cell effector function are advantageous to prevent auto-reactivity, which leads to autoimmune disorders, but in the context of cancer it can prevent T cell recognition of cancerous cells and limit tumor cell clearance. Clinical success using ipilimumab, a monoclonal antibody against CTLA-4, for the treatment of metastatic melanoma led to the first Food and Drug Administration (FDA) approval of an immune checkpoint inhibitor in 2011 [3]. Another major target of immune checkpoint inhibition is programmed cell death protein 1 (PD-1). PD-1 is also an inhibitory molecule expressed on the surface of immune cells, and when bound to its ligand PD-L1 (programmed death-ligand 1), it initiates downstream signaling events that cause decreased T cell activation, proliferation, survival, and cytokine production. The FDA approved the use of the PD-1 inhibitors nivolumab and pembrolizumab in 2014 [3–6]. Since 2014, several additional immune checkpoint inhibitors (ICIs) have received FDA-approval, and their applicability has expanded to a variety of different cancers due to their efficacy (Table 1). The utilization of ICIs to treat solid tumors has significantly improved patient outcomes. Treatment with ICIs has been associated with significant survival benefits for most cancer types for which they have been approved [7–9]. For example, in metastatic melanoma the 5-year overall survival rate improved from just 5% to roughly 50% following the approval of combined treatment with ipilimumab and nivolumab [10–13]. Additionally, treatment with ICIs is associated with a more durable response [8,9,11,13,14]. For these reasons, ICIs are now used as the first line of treatment for numerous cancers.

Despite the great successes of immune checkpoint inhibition, obstacles remain. Some patients do not respond to ICIs, and others develop resistance. Across all cancers for which immune checkpoint inhibition has been approved, it is estimated that only ~20% of patients respond to ICIs [14]. Therefore, it is crucial to understand the mechanisms that confer both innate and acquired resistance in order to develop new approaches to improve responsiveness to immune checkpoint inhibition. Both tumor cell-intrinsic and -extrinsic factors mediate resistance to ICIs. Tumor cell-intrinsic factors that lead to resistance include low tumor mutational burden and enhanced oncogenic signaling [15,16]. Tumor-extrinsic factors can also limit response to ICIs. The extracellular matrix (ECM) and stroma surrounding the tumor can prevent T cell infiltration into the tumor, and an abundance of immunosuppressive immune cells can hinder T cell infiltration and function [17–19]. Based on this information, ICIs have also been tested in combination with other therapies, including chemotherapy, radiotherapy, and targeted therapies, to improve response [20]. Here, we discuss the various roles of focal adhesion kinase (FAK) in regulating the tumor microenvironment (TME) and the clinical potential for combining immune checkpoint inhibition with targeted therapies against FAK.

## Focal Adhesion Kinase (FAK)

### FAK structure and function

**Figure 1.**
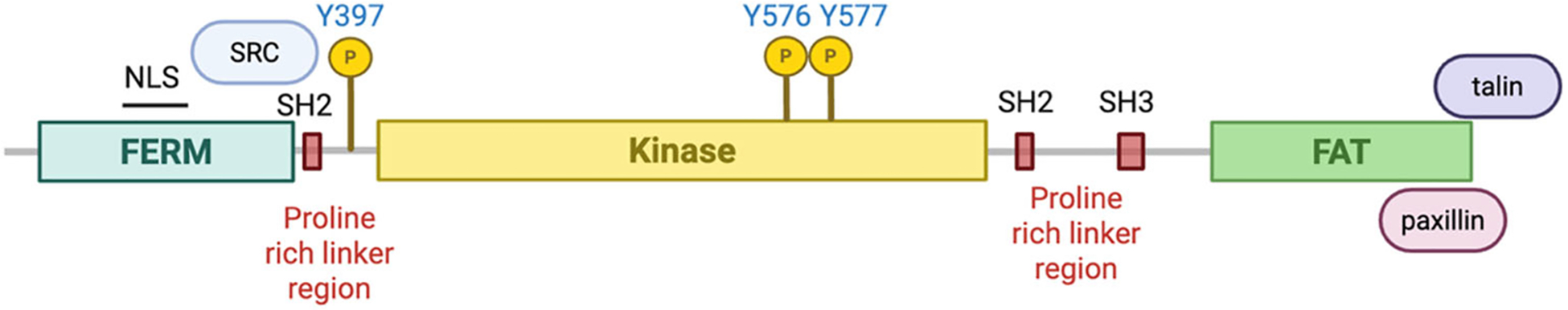
FAK protein structure and key residues for activation. Schematic includes key functional domains and phosphorylation sites. FERM: 4.1 Ezrin, Radixin, Moesin Homology; FAT: Focal Adhesion Targeting; NLS: Nuclear Localization Sequence; SH2: Src Homology 2; SH3: Src Homology 3. Created with Biorender.com.

FAK, encoded by the *PTK2* gene, is a ubiquitously expressed non-receptor tyrosine kinase involved in numerous oncogenic signaling pathways [17]. Canonically, FAK signals downstream of integrins and growth factor receptors at focal adhesions to stimulate cell migration and invasion. FAK consists of three protein domains: the N-terminal 4.1 ezrin, radixin, moesin homology (FERM) domain, the kinase domain, and the C-terminal focal adhesion targeting (FAT) domain (Figure 1). Proline-rich linker regions separate each domain, providing binding sites for Src homology 2 (SH2) and Src homology 3 (SH3) domain-containing proteins. The FERM domain is highly conserved and is primarily responsible for localization to the plasma membrane and binding to lipids and membrane proteins. The FERM domain also serves to regulate FAK through autoinhibitory interactions with its kinase domain, which blocks access to Y397, a FAK autophosphorylation site critical for its activation [21–23]. External stimuli such as lipid binding, binding to the ECM, and integrin signaling induces a conformational change that relieves the autoinhibitory interaction of the FERM and kinase domains, promoting autophosphorylation of Y397 and creating a SH2-domain binding site. Src recruitment then facilitates phosphorylation of Y576 and Y577 within the activation loop of the kinase domain, activating FAK for downstream signaling [18,21–25]. The FAT domain is required for localization to focal adhesions through direct binding with other focal adhesion proteins like talin and paxillin [18,21,22].

In addition to its roles in cell adhesion and migration, FAK has several reported functions that contribute to cancer progression, including promoting cell survival, proliferation, cancer cell stemness, epithelial to mesenchymal transition (EMT), and chemotherapeutic resistance [17–19,26–34]. Clinical data demonstrate that FAK overexpression in a number of different cancers, including ovarian, breast, pancreatic, lung, and colorectal cancer is correlated with poor prognosis [18,22,26,35–38]. These numerous and well-documented oncogenic functions of FAK have made it an attractive therapeutic target, and multiple pharmacologic agents have been developed and tested in the pre-clinical and clinical setting. As a single agent, FAK inhibitors have demonstrated acceptable safety profiles but have had limited success [17,39–41]. It has been suggested that FAK inhibitors as a monotherapy may primarily affect the adhesion and migratory roles of FAK, which could be insufficient to control cancer progression [21]. Instead, like most targeted therapies, FAK inhibition may have greater clinical benefit in combination with other agents. A better mechanistic understanding of how FAK exerts its various pro-tumorigenic functions can provide insight on the types of combinations that will be most advantageous. Recent research on FAK has uncovered non-canonical functions, including its regulation of the TME warranting further investigation on co-targeting FAK to boost ICI response.

### Effects of FAK on T cell signaling within TME

As current immune checkpoint inhibition strategies rely on releasing the “brakes” on T cell signaling to promote tumor cell clearance, it is critical to understand how the addition of a targeted therapy may alter T cell function. FAK is expressed at low levels in human T cells and has been shown to negatively regulate TCR function in activated CD4^+^ T cells. TCR signaling involves multiple phosphorylation events. Upon TCR stimulation, constitutively active lymphocyte-specific protein tyrosine kinase (LCK) is recruited to the TCR to initiate phosphorylation of zeta-chain-associated protein kinase 70 (ZAP-70) and adaptor proteins linker for activation of T cells (LAT) and SH2 domain containing leukocyte protein of 76kDa (SLP-76). LAT and SLP-76 activate effector proteins phospholipase C-γ1 (PLC-γ1) and protein kinase B (AKT) to facilitate downstream signaling [42,43]. Selective suppression of FAK through FAK-specific miRNAs in both Jurkat and activated human CD4^+^ T cells enhanced phosphorylation of LAT, SLP-76, PLC-γ1 and AKT. FAK-deficient T cells also displayed increased antigen sensitivity. When stimulated with low doses of anti-CD3 antibody, FAK-deficient T cells produce more interleukin (IL)-2 and interferon (IFN)-γ and increase CD69 surface expression [42,43]. One way in which FAK has been shown to suppress TCR signaling is through the recruitment of C-terminal Src kinase (CSK), a tyrosine kinase that suppresses Src family kinases. FAK association with CSK leads to inhibitory phosphorylation of LCK at Y505, suppressing LCK function. In FAK-deficient T cells, CSK membrane association is decreased and levels of LCK Y505 phosphorylation are lower [21,42,43]. FAK can also prevent activation of naïve T cells by prompting the dissociation of dendritic cell-T cell conjugation. Prolonged dendritic cell-T cell conjugation supports T cell fitness and activation [21,44]. These data demonstrate the ability of FAK to negatively regulate T cell activation, but whether FAK regulates T cell differentiation and memory is unknown.

In preclinical models of cancer, FAK has been shown to reduce intratumoral T cell infiltration and promote T cell exhaustion, dampening immune responses. In late-stage high-grade serous ovarian cancer (HGSOC), active FAK is significantly increased and is correlated with elevated levels of CD155, a ligand for the immune checkpoint protein TIGIT. High FAK expression in patient samples also correlates with lower levels of CD3^+^ tumor-infiltrating lymphocytes. Using an aggressive mouse model of ovarian cancer, Ozmadenci *et al*. tested pharmacological FAK inhibition and observed a decrease in tumor burden and CD155 levels, as well as an increase in tumor-infiltrating lymphocytes. Combining the FAK inhibitor with TIGIT-blocking antibodies resulted in a reduction in tumor burden and in Tregs, with an increase in immune cell activation and prolonged survival [45]. In triple-negative breast cancer (TNBC), FAK mRNA expression is correlated with expression of the PD-1 ligand, PD-L1, and FAK inhibition improved response to PD-L1-neutralizing antibodies [17,46]. This relationship between FAK, PD-L1 expression, and response to immune checkpoint inhibition was also observed in a model of hepatocellular carcinoma (HCC) [47]. While these studies suggest FAK inhibition can boost response to ICI through upregulation of checkpoint ligands, the specific mechanisms behind FAK-mediated checkpoint ligand expression are unclear.

In addition to promoting immune checkpoint ligand expression, FAK also regulates chemokine and cytokine transcription and secretion to alter T cell recruitment and activity. A study in squamous cell carcinoma (SCC) revealed that active FAK recruits Tregs and inhibits cytotoxic CD8^+^ T cell infiltration through upregulation of chemokines and cytokines. Specifically, FAK translocates to the nucleus to promote gene expression of IL-33 by altering chromatin accessibility. IL-33 is retained in the nucleus to promote transcription of the soluble ST2 (sST2) receptor. sST2 has been termed a “decoy” receptor for secreted IL-33, blocking CD8^+^ T cell recruitment and activation [48,49]. While this increase in chemokine and cytokine transcription has been shown to be dependent on nuclear FAK kinase activity, the key mediators of this process and how they functionally interact with each other is unknown.

Another nuclear function of FAK in T cell signaling is suppression of antigen processing and presentation [50]. In a mouse model of pancreatic ductal carcinoma (PDAC), FAK deletion in PDAC cells through CRISPR/Cas9 gene-editing reduced tumor growth and enhanced CD8^+^ T cell infiltration. This response was mediated through the elevated expression of the immunoproteasome, causing an increase in antigen diversity and antigen presentation on the surface of the cell through upregulation of major histocompatibility complex-I (MHC-I) [50]. Notably, treatment with a catalytic FAK inhibitor did not have a significant effect on antigen processing and presentation, suggesting this occurs through a kinase-independent mechanism [50]. This demonstrates the need to clearly identify which functions of FAK are kinase-dependent or -independent to inform alternative drug designs for targeting FAK. Taken together, the reported functions of FAK within and on T cells in the TME suggest that the combination of FAK inhibition with ICIs may be an effective therapeutic strategy.

### Effects of FAK on the stroma of the TME

The TME is made up of more than just tumor and surrounding immune cells. It is also comprised of stromal cells, secreted factors, the ECM, and blood and lymphatic vessels [17,51]. Each component can influence the immune response. For example, the formation of new blood vessels through angiogenesis can lead to immunosuppression. In cancer, there is often an excessive number of blood vessels, which limit the efficacy of immune checkpoint inhibition. These vessels are often disorganized, lacking structural hierarchy [19]. Additionally, they have poor cell-to-cell contacts, making them “leaky.” These immature blood vessels leak fluid, creating high interstitial pressure that can collapse blood vessels, limiting the delivery of oxygen and nutrients. This dysregulation also hinders immune cell infiltration and drug delivery [19,52,53]. Thus, anti-angiogenics have been explored as a combination therapy with ICIs. Most anti-angiogenics target vascular endothelial growth factor (VEGF), and the use of these agents with immune checkpoint therapy has been FDA-approved in multiple cancer types [53,54].

Interestingly, FAK is essential for angiogenesis. In mouse models, global FAK knockout or overexpression of kinase-dead FAK is embryonic lethal due to impairments in endothelial cell (EC) proliferation, survival, and polarity, which causes defects in blood vessel formation [18,55]. EC-specific knockout of FAK has similar effects on blood vessel formation and lethality [18,56,57]. In adult mice, FAK inhibition decreases angiogenesis [18,58,59]. This demonstrates that FAK plays a crucial role in angiogenesis in normal development. In the context of cancer, tumor-associated ECs have elevated levels of FAK mRNA and protein [18,60,61]. In a mouse model of melanoma, depleting EC FAK expression through tamoxifen treatment of *Pdgfb*-iCreER;FAK^fl/fl^ mice was shown to suppress tumor growth and prevent tumor angiogenesis by inhibiting VEGF-induced AKT phosphorylation [59]. The ability of FAK to mediate its effects on angiogenesis requires FAK catalytic activity. Investigators blocked FAK activation in ECs through mutation of the Y397 autophosphorylation site in EC-CRE+;FAK^Y397F/Y397F^-mutant mice and challenged these mice with B16F0 melanoma cells. The researchers observed a reduction in tumor growth and angiogenesis [18,62]. The loss of kinase activity inhibited VEGF-stimulated EC proliferation, survival, and migration [62]. FAK has also been demonstrated to facilitate blood vessel formation through promoting vascular endothelial growth factor receptor 2 (VEGFR2) expression in a kinase-dependent manner [58].

Another hallmark of angiogenesis is increased vascular permeability, which is controlled by cell-cell adhesions between ECs [18,63]. FAK has a critical role in VEGF-stimulated vascular permeability, in which it facilitates cellular junction breakdown through binding to VE-cadherin and phosphorylation of β-catenin. Phosphorylation of β-catenin by FAK causes VE-cadherin-β-catenin complex dissociation and VE-cadherin internalization [19,64]. Inhibiting FAK either genetically or pharmacologically limits EC vascular permeability [64]. Collectively, these data support targeting FAK to inhibit angiogenesis, which can bolster response to ICI treatments. FAK inhibitors have already been reported as potent anti-angiogenic agents and have been shown to reduce tumor angiogenesis in animal models of colon, ovarian, and hepatocellular carcinoma [62,65,66].

Another major component of the TME is the ECM. The ECM comprises up to 60% of the tumor mass and cancer-associated fibroblasts (CAFs) are a major source of ECM molecules [67]. Excessive production of ECM molecules like collagen creates a fibrotic and desmoplastic microenvironment that is correlated with poor prognosis in cancers like breast cancer, gastric cancer, pancreatic cancer, and lung cancer [67]. The increased density and stiffness of the ECM can limit the efficacy of immune checkpoint inhibition by creating a physical barrier that limits immune cell infiltration and delivery of systemic immunotherapy. Additionally, the fibrotic stroma reduces diffusion of oxygen and other nutrients, causing hypoxia. Hypoxia upregulates the expression of immunosuppressive factors, preventing effector function. Fibrosis is correlated with worse responses to immune checkpoint inhibition, sparking interest in combining ECM-targeting agents with immunotherapies [67]. FAK has been implicated in promoting fibrosis in some contexts. In lung fibrosis patients, enhanced activation of FAK in fibroblasts is commonly observed. In a mouse model of lung fibrosis, fibrosis was induced through intratracheally instilled bleomycin and mice were treated with the catalytic FAK inhibitor PF-562,271 or with siRNAs targeting FAK. The treatment results showed a marked reduction in fibrosis when inhibiting FAK pharmacologically or genetically [68].

FAK has also been shown to induce a fibrotic TME in cancer, limiting immune cell infiltration and dampening response to immune checkpoint inhibition. PDAC is a highly desmoplastic cancer and responds poorly to ICIs. FAK is activated in patient-derived PDAC tumors and stromal compartments and is correlated with high levels of fibrosis and limited CD8^+^ cytotoxic T cell infiltration [26,69]. In an orthotopic mouse model of PDAC, Stokes *et al*. found that FAK inhibition with the small molecule inhibitor PF-562,271 decreased total CAF numbers and reduced tumor growth. Researchers proposed that FAK inhibition limits tumor growth through blocking CAF recruitment and/or proliferation [69]. Using the KPC mouse model of PDAC, which is not responsive to immunotherapy, Jiang *et al*. tested the effects of FAK inhibition alone or in combination with ICIs on tumor progression, fibrosis, and immune cell infiltration. They found that single-agent FAK inhibition with the small molecule inhibitor VS-4718 decreased fibrosis, demonstrated by a decrease in collagen deposition and reduction in fibroblasts expressing fibroblast activation protein α (FAPα) and α-smooth muscle actin (α-SMA) [26]. They also determined that FAK inhibition decreased fibroblast proliferation. The reduction in fibrosis led to a decrease in immunosuppressive cell populations such as Tregs. Using short hairpin RNA (shRNA) against FAK, the researchers also tested the effect of FAK-knockdown in an orthotopic model of KPC. They observed similar results on tumor growth, fibrosis, and immune infiltration, including a significant increase in CD8^+^ T cell infiltration. Combining FAK inhibition with immune checkpoint inhibition rendered the previously unresponsive PDAC tumors sensitive to therapy, noting elevated CD8^+^ T cell and reduced Treg infiltration within the tumor [26]. Similar findings have been observed in a mouse model of non-small cell lung cancer (NSCLC), in which FAK inhibition renders this immunologically “cold” tumor responsive to immune checkpoint inhibition through a decrease in fibrosis and increase in effector T cell infiltration [70]. These studies highlight the role of FAK in promoting fibrosis to create an immunosuppressive TME and indicate that targeting FAK could promote immune surveillance and overcome resistance to immune checkpoint inhibition.

## Conclusions and Future Directions

### Clinical potential of FAK inhibition and immunotherapy

In summary, ICIs have improved cancer care but have limited patient response. Efficacy of immune checkpoint inhibition may be improved when combined with targeted therapies against immunosuppressive signaling pathways. FAK has emerged as a promising therapeutic target for this purpose, as it is intricately involved in numerous signaling pathways that promote a pro-tumorigenic, immunosuppressive TME. Particularly, FAK is essential for VEGF-mediated angiogenesis, a pathway that has already been successfully targeted clinically with ICIs [53]. Additionally, FAK promotes the establishment of a fibrotic microenvironment, which limits responses to immunotherapy. Further, FAK functions within T cells to negatively regulate their activity though inhibition of TCR signaling. FAK also functions within tumor cells to promote immune evasion by increasing cell surface expression of immune checkpoint ligands and decreasing antigen presentation. Reversing these effects through FAK inhibition may unleash a greater immune response when treated with ICIs.

Numerous pre-clinical studies have already demonstrated enhanced response from the combination of ICIs and FAK inhibitors. Additionally, multiple FAK inhibitors demonstrate acceptable safety profiles in early-stage clinical trials [17,39–41]. Currently, no FAK inhibitors are FDA-approved as a single-agent therapy due to their modest clinical success. This modest efficacy may be due to activation of other signaling pathways, which is a common resistance mechanism to single-target inhibitors. An alternative explanation is that the FAK inhibitors currently in clinical testing only target FAK catalytic activity. As outlined in this review, FAK possesses multiple non-catalytic functions that contribute to cancer progression. To this end, numerous non-catalytic FAK inhibitors have been developed and tested pre-clinically. Proteolysis targeting chimeras (PROTACs), which selectively target proteins for degradation, are actively being explored as a way to block both FAK catalytic and non-catalytic functions through FAK degradation. At least three PROTACs targeting FAK are being studied pre-clinically and some have displayed greater efficacy than catalytic FAK inhibitors [71,72]. The development of new pharmacologic agents that dually target the FAK kinase-dependent and kinase-independent functions or facilitate FAK degradation may yield a greater clinical response.

Although FAK inhibitors have not yet been successful as single agent therapies, they have been effective in combination with other treatments. Recently, the catalytic FAK inhibitor defactinib was granted accelerated approval by the FDA for the treatment of KRAS-mutant low grade serious ovarian cancer (LGSOC) in combination with the RAF/MEK clamp avutometinib [73]. Pre-clinical studies have highlighted the potential for FAK inhibition in more complex combination strategies with immunotherapy while maintaining tolerability. For instance, in a mouse model of melanoma that does not typically respond to immune checkpoint therapy, treatment with RAF, MEK, and FAK inhibitors alongside immune checkpoint inhibition significantly prolongs overall survival [74]. The potential of co-targeting FAK and immune checkpoints is gaining traction, with multiple clinical trials testing this combination underway (Table 2). Ongoing trials are testing the combination of FAK inhibition and immune checkpoint inhibition primarily in pancreatic cancers, but this strategy should be applicable to a range of cancer types, particularly those discussed in this review. While PD-1 and CTLA-4 have been the primary targets of immune checkpoint inhibition, additional immune checkpoints continue to be evaluated. Currently, immune checkpoints lymphocyte-activation gene 3 (LAG-3), T cell immunoglobulin and mucin-domain containing-3 (TIM-3), and V-domain Ig suppressor of T cell activation (VISTA) are actively being pursued as targets for immunotherapy [75]. Although their signaling mechanisms are distinct, their pathways share a similar immunosuppressive effect on T cells that dampen their function. Current research on the crosstalk between these immune checkpoints with FAK is lacking, but the various mechanisms by which FAK inhibition enhances response to immune checkpoint inhibition by altering T cell function and the tumor stroma likely applies to other immune checkpoint targets. Outside of its effects on the immune system, there may be additional benefit for including FAK inhibition in combination strategies, as it is a key mediator of several oncogenic processes, including cancer cell survival, proliferation, stemness, metastasis, and therapeutic resistance.

This review focuses on FAK regulation of the TME as it relates to T cell function and stromal reprogramming to alter response to immune checkpoint inhibition, as T cells are the primary target of ICIs and these drugs have been successfully combined with anti-angiogenics [53]. Although our understanding of FAK regulation on T cell-mediated antitumor immunity is growing, large gaps remain. For example, how does FAK mediate the expression of checkpoint ligands such as CD155 and PD-L1? How does FAK modify chromatin architecture to regulate gene expression of chemokines and cytokines? How does FAK affect T cell differentiation and memory? Is there crosstalk between FAK signaling and other immune checkpoint signaling pathways, and can they be co-targeted? These questions serve as a guide for continued research as we pursue the goal of combined FAK and immune checkpoint inhibition.

In addition to T cells, FAK also regulates other immune cells to create an immunosuppressive TME, including macrophages and neutrophils, and some of these functions have been reviewed [19]. The immunosuppressive role of FAK in multiple immune cell populations supports the strategy of co-targeting FAK alongside ICIs to stimulate a greater immune response. More research on the functions of FAK in the various immune cell populations of the TME may provide insight for novel combinations of FAK inhibitors and immunomodulatory agents. It should be noted that conflicting roles of FAK on the TME have been reported in specific cell types. For instance, Lechertier *et al*. found that FAK inhibition in pericytes can induce angiogenesis and tumor growth by stimulating Gas6/Axl signaling in melanoma, lung, and pancreatic models [76]. However, in an ovarian cancer model, Halder *et al*. reported that FAK inhibition reduced tumor burden and suppressed angiogenesis in part by blocking pericyte production of VEGF and lowering pericyte coverage of the tumor [66]. This suggests that the reported functions of FAK may be context-dependent and highlights the importance of validating findings across different cell and cancer types to identify which patients are most likely to benefit from FAK inhibition.

The development of predictive biomarkers may also inform patient selection. Currently, there are no established biomarkers to predict response to FAK inhibitors. However, copy number gains of *PTK2*, the gene encoding FAK, has been shown to be a predictive marker for sensitivity to FAK inhibition in a breast cancer cell line, and *PTK2* gene amplifications confer worse prognosis in patients with various cancer types [35]. This suggests that *PTK2* copy number could be used as a guide for patient selection. E-cadherin has also been proposed as a potential biomarker, as its expression has been correlated with resistance to FAK inhibitors in Merlin-deficient mesothelioma cells [72,77]. Future research should seek to identify and validate potential biomarkers for response and resistance not just for FAK inhibition but for co-targeting FAK and immune checkpoints, to maximize patient benefit.

**Figure 2.**
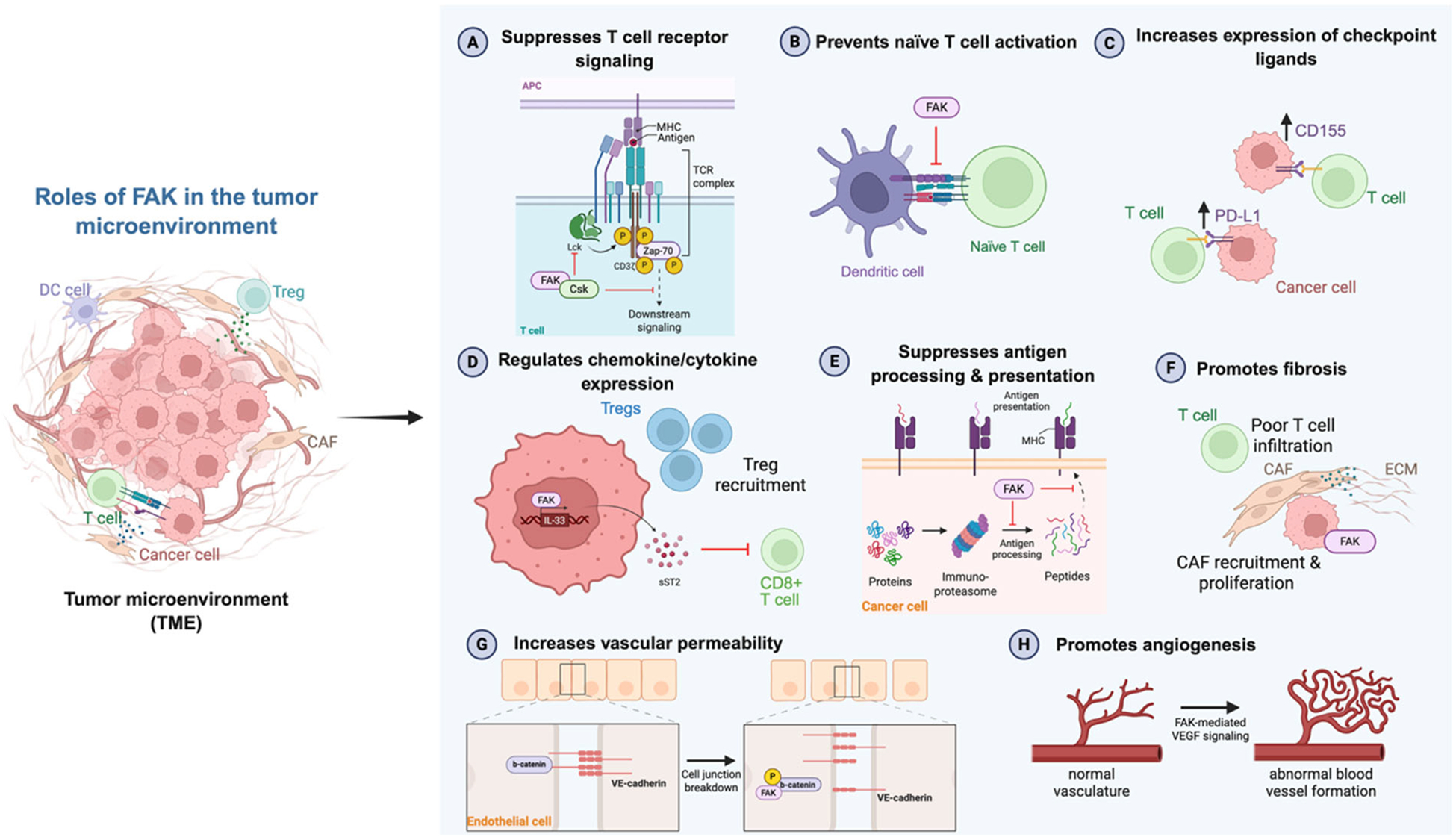
Roles of FAK in the tumor microenvironment. FAK functions in the tumor microenvironment to regulate T cell activity and stromal characteristics. APC: Antigen Presenting Cell; CAF: Cancer-Associated Fibroblast; DC: Dendritic Cell; ECM: Extracellular Matrix; MHC: Major Histocompatibility Complex; TCR: T cell Receptor; TME: Tumor Microenvironment; Treg: Regulatory T cell. Created with BioRender.com.

Finally, while not discussed at length here, FAK mediates its effects on the TME through both cytoplasmic and nuclear signaling, and through kinase-dependent and -independent mechanisms (Figure 2 and Table 3). As we continue to reveal more about FAK signaling and regulation of the TME, delineating the precise signaling mechanisms at play will be informative for the development of drug design and treatment strategies.

## Figures and Tables

**Table 1.** Current FDA-approved immune checkpoint inhibitors. Drugs@FDA: FDA-Approved Drugs.

Drug (Brand Name)	Year of Approval	Target	Cancer Type
Ipilimumab	2011	CTLA-4	Melanoma, Renal cell carcinoma (RCC), Colorectal cancer, Hepatocellular carcinoma (HCC), Non-small cell lung cancer (NSCLC), Malignant pleural mesothelioma (MPM), Esophageal cancer
Pembrolizumab	2014	PD-1	Melanoma, NSCLC, MPM, Head and neck squamous cell cancer (HNSCC), Classical Hodgkin Lymphoma (cHL), Primary Mediastinal Large B-cell lymphoma (PMBCL), Urothelial cancer, Microsatellite Instability-High or Mismatch Repair Deficient Cancer (MSI-H/dMMR), Gastric cancer, Esophageal cancer, Cervical cancer, HCC, Biliary tract cancer (BTC), Merkel cell carcinoma (MCC), RCC, Endometrial carcinoma, Tumor Mutational Burden-High Cancer (TMB-H), Squamous Cell carcinoma (SCC), Triple-Negative Brest Cancer (TNBC)
Nivolumab	2014	PD-1	RCC, melanoma, NSCLC, SCC, Urothelial cancer (UC), Colorectal cancer, HCC, Esophageal cancer, Gastric cancer
Atezolizumab	2016	PD-L1	NSCLC, Small cell lung cancer (SCLC), HCC, melanoma, Alveolar soft part sarcoma (ASPS)
Avelumab	2017	PD-L1	MCC, UC, RCC
Durvalumab	2017	PD-L1	NSCLC, SCLC, BTC, HCC, Muscle invasive bladder cancer (MIBC), Endothelial cancer
Cemiplimab	2018	PD-1	SCC, Basal cell carcinoma (BCC), NSCLC
Dostarlimab	2021	PD-1	Endometrial, dMMR recurrent or advanced solid tumors
Relatlimab	2022	LAG-3	Melanoma
Retifanlimab	2023	PD-1	SCC, MCC

CTLA-4: Cytotoxic T-lymphocyte-Associated Protein 4; dMMR: Deficient DNA Mismatch Repair; LAG-3: Lymphocyte-Activation Gene 3; PD-1: Programmed Cell Death Protein 1; PD-L1: Programmed Death-Ligand 1

**Table 2.** Clinical trials testing combined FAK inhibition and immune checkpoint inhibition. Clinicaltrials.gov.

Trial ID/Name	FAK inhibitor	Combination	Cancer Type(s)	Phase
NCT02758587 (FAK-PD1)	Defactinib	Pembrolizumab (anti-PD-1)	Advanced solid tumors; expansions in PDAC, NSCLC, mesothelioma	I/II
NCT02546531	Defactinib	Pembrolizumab (anti-PD-1) + gemcitabine	Advanced pancreatic cancer	I
NCT03727880	Defactinib	Pembrolizumab (anti-PD-1)	PDAC	II

FAK: Focal Adhesion Kinase; PD-1: Programmed Cell Death Protein 1; PDAC: Pancreatic Ductal Carcinoma; NSCLC: Non-Small Cell Lung Cancer

**Table 3.** FAK functions by cellular location and kinase activity.

FAK Function	Kinase-dependent	Kinase-independent
Elevate chemokine/cytokine gene expression	**X**	
Promote immune checkpoint ligand expression	**X**	
Suppress tumor cell antigen processing and presentation		**X**
Reduce TCR signaling	**X**	**X**
Promote blood vessel formation	**X**	
Increase vascular permeability	**X**	
Induce CAF proliferation and recruitment	**X**	

CAF: Cancer-Associated Fibroblast; FAK: Focal Adhesion Kinase; TCR: T cell Receptor

## References

[1] Zhang Y, Zhang Z. The history and advances in cancer immunotherapy: understanding the characteristics of tumor-infiltrating immune cells and their therapeutic implications. Cell Mol Immunol. 2020 Aug;17(8):807–21. 32612154 10.1038/s41423-020-0488-6 PMC7395159

[2] Raghani NR, Chorawala MR, Mahadik M, Patel RB, Prajapati BG, Parekh PS. Revolutionizing cancer treatment: comprehensive insights into immunotherapeutic strategies. Med Oncol. 2024 Jan 9;41(2):51. 38195781 10.1007/s12032-023-02280-7

[3] Buchbinder EI, Desai A. CTLA-4 and PD-1 Pathways. Am J Clin Oncol. 2016 Feb;39(1):98–106. 26558876 10.1097/COC.0000000000000239 PMC4892769

[4] Patsoukis N, Wang Q, Strauss L, Boussiotis VA. Revisiting the PD-1 pathway. Sci Adv. 2020 Sep 18;6(38):eabd2712. 32948597 10.1126/sciadv.abd2712 PMC7500922

[5] Han Y, Liu D, Li L. PD-1/PD-L1 pathway: current researches in cancer. Am J Cancer Res. 2020 Mar 1;10(3):727–42. 32266087 PMC7136921

[6] Sharpe AH, Pauken KE. The diverse functions of the PD1 inhibitory pathway. Nat Rev Immunol. 2018 Mar;18(3):153–67. 28990585 10.1038/nri.2017.108

[7] Miller SR, Schipper M, Fritsche LG, Jiang R, Strohbehn G, Ötleş E, Pan-Cancer Survival Impact of Immune Checkpoint Inhibitors in a National Healthcare System. Cancer Med. 2024 Nov 7;13(21):e70379. 39508134 10.1002/cam4.70379 PMC11541111

[8] Pons-Tostivint E, Cartouche A, Vaflard P, Ricci F, Loirat D, Hescot S, Comparative Analysis of Durable Responses on Immune Checkpoint Inhibitors Versus Other Systemic Therapies: A Pooled Analysis of Phase III Trials. JCO Precis Oncol. 2019 Feb;(3):1–10. 10.1200/PO.18.00114 35100670

[9] Brahmer JR, Lee JS, Ciuleanu TE, Bernabe Caro R, Nishio M, Urban L, Five-Year Survival Outcomes With Nivolumab Plus Ipilimumab Versus Chemotherapy as First-Line Treatment for Metastatic Non–Small-Cell Lung Cancer in CheckMate 227. J Clin Oncol. 2023 Feb 20;41(6):1200–12. 36223558 10.1200/JCO.22.01503 PMC9937094

[10] Hassel JC, Zimmer L, Sickmann T, Eigentler TK, Meier F, Mohr P, Medical Needs and Therapeutic Options for Melanoma Patients Resistant to Anti-PD-1-Directed Immune Checkpoint Inhibition. Cancers. 2023 Jun 30;15(13):3448. 37444558 10.3390/cancers15133448 PMC10341224

[11] Wolchok JD, Chiarion-Sileni V, Gonzalez R, Grob JJ, Rutkowski P, Lao CD, Long-Term Outcomes With Nivolumab Plus Ipilimumab or Nivolumab Alone Versus Ipilimumab in Patients With Advanced Melanoma. J Clin Oncol. 2022 Jan 10;40(2):127–37. 34818112 10.1200/JCO.21.02229 PMC8718224

[12] Sabbatino F, Liguori L, Pepe S, Ferrone S. Immune checkpoint inhibitors for the treatment of melanoma. Expert Opin Biol Ther. 2022 May;22(5):563–76. 35130816 10.1080/14712598.2022.2038132 PMC9038682

[13] Kahlon N, Doddi S, Yousif R, Najib S, Sheikh T, Abuhelwa Z, Melanoma Treatments and Mortality Rate Trends in the US, 1975 to 2019. JAMA Netw Open. 2022 Dec 6;5(12):e2245269. 36472871 10.1001/jamanetworkopen.2022.45269 PMC9856246

[14] Haslam A, Olivier T, Prasad V. How many people in the US are eligible for and respond to checkpoint inhibitors: An empirical analysis. Int J Cancer. 2025;156(12):2352–9. 39887747 10.1002/ijc.35347

[15] Wu B, Zhang B, Li B, Wu H, Jiang M. Cold and hot tumors: from molecular mechanisms to targeted therapy. Signal Transduct Target Ther. 2024 Oct 18;9(1):1–65. 39420203 10.1038/s41392-024-01979-x PMC11491057

[16] Li J, Byrne KT, Yan F, Yamazoe T, Chen Z, Baslan T, Tumor cell-intrinsic factors underlie heterogeneity of immune cell infiltration and response to immunotherapy. Immunity. 2018 Jul 17;49(1):178–93.e7. 29958801 10.1016/j.immuni.2018.06.006 PMC6707727

[17] Murphy JM, Rodriguez YAR, Jeong K, Ahn EYE, Lim STS. Targeting focal adhesion kinase in cancer cells and the tumor microenvironment. Exp Mol Med. 2020 Jun 9;52(6):877–86. 32514188 10.1038/s12276-020-0447-4 PMC7338452

[18] Chuang HH, Zhen YY, Tsai YC, Chuang CH, Hsiao M, Huang MS, FAK in Cancer: From Mechanisms to Therapeutic Strategies. Int J Mol Sci. 2022 Jan;23(3):1726. 35163650 10.3390/ijms23031726 PMC8836199

[19] Sulzmaier FJ, Jean C, Schlaepfer DD. FAK in cancer: mechanistic findings and clinical applications. Nat Rev Cancer. 2014 Sep;14(9):598–610. 25098269 10.1038/nrc3792 PMC4365862

[20] Barbari C, Fontaine T, Parajuli P, Lamichhane N, Jakubski S, Lamichhane P, Immunotherapies and Combination Strategies for Immuno-Oncology. Int J Mol Sci. 2020 Jul 15;21(14):5009. 32679922 10.3390/ijms21145009 PMC7404041

[21] Dawson JC, Serrels A, Stupack DG, Schlaepfer DD, Frame MC. Targeting FAK in anticancer combination therapies. Nat Rev Cancer. 2021 May;21(5):313–24. 33731845 10.1038/s41568-021-00340-6 PMC8276817

[22] Lietha D, Cai X, Ceccarelli DFJ, Li Y, Schaller MD, Eck MJ. Structural Basis for the Autoinhibition of Focal Adhesion Kinase. Cell. 2007 Jun 15;129(6):1177–87. 17574028 10.1016/j.cell.2007.05.041 PMC2077847

[23] Frame MC, Patel H, Serrels B, Lietha D, Eck MJ. The FERM domain: organizing the structure and function of FAK. Nat Rev Mol Cell Biol. 2010 Nov;11(11):802–14. 20966971 10.1038/nrm2996

[24] Acebrón I, Righetto RD, Schoenherr C, de Buhr S, Redondo P, Culley J, Structural basis of Focal Adhesion Kinase activation on lipid membranes. EMBO J. 2020 Oct;39(19):e104743. 32779739 10.15252/embj.2020104743 PMC7527928

[25] Calalb MB, Polte TR, Hanks SK. Tyrosine phosphorylation of focal adhesion kinase at sites in the catalytic domain regulates kinase activity: a role for Src family kinases. Mol Cell Biol. 1995 Feb;15(2):954–63. 7529876 10.1128/mcb.15.2.954 PMC231984

[26] Jiang H, Hegde S, Knolhoff BL, Zhu Y, Herndon JM, Meyer MA, Targeting focal adhesion kinase renders pancreatic cancers responsive to checkpoint immunotherapy. Nat Med. 2016 Aug;22(8):851–60. 27376576 10.1038/nm.4123 PMC4935930

[27] Lee BY, Timpson P, Horvath LG, Daly RJ. FAK signaling in human cancer as a target for therapeutics. Pharmacol Ther. 2015 Feb 1;146:132–49. 25316657 10.1016/j.pharmthera.2014.10.001

[28] Zhou J, Yi Q, Tang L. The roles of nuclear focal adhesion kinase (FAK) on Cancer: a focused review. J Exp Clin Cancer Res. 2019 Dec;38(1):250. 31186061 10.1186/s13046-019-1265-1 PMC6560741

[29] Cance WG, Golubovskaya VM. Focal Adhesion Kinase Versus p53: Apoptosis or Survival? Sci Signal. 2008 May 20;1(20):pe22. 18493017 10.1126/stke.120pe22 PMC4560103

[30] Mitra SK, Hanson DA, Schlaepfer DD. Focal adhesion kinase: in command and control of cell motility. Nat Rev Mol Cell Biol. 2005 Jan;6(1):56–68. 15688067 10.1038/nrm1549

[31] Li XY, Zhou X, Rowe RG, Hu Y, Schlaepfer DD, Ilić D, Snail1 controls epithelial–mesenchymal lineage commitment in focal adhesion kinase–null embryonic cells. J Cell Biol. 2011 Nov 28;195(5):729–38. 22105351 10.1083/jcb.201105103 PMC3257570

[32] Fan H, Zhao X, Sun S, Luo M, Guan JL. Function of Focal Adhesion Kinase Scaffolding to Mediate Endophilin A2 Phosphorylation Promotes Epithelial-Mesenchymal Transition and Mammary Cancer Stem Cell Activities in Vivo. J Biol Chem. 2013 Feb 1;288(5):3322–33. 23255596 10.1074/jbc.M112.420497 PMC3561552

[33] Canel M, Serrels A, Frame MC, Brunton VG. E-cadherin–integrin crosstalk in cancer invasion and metastasis. J Cell Sci. 2013 Jan 15;126(2):393–401. 23525005 10.1242/jcs.100115

[34] Luo M, Zhao X, Chen S, Liu S, Wicha MS, Guan JL. Distinct FAK activities determine progenitor and mammary stem cell characteristics. Cancer Res. 2013 Sep 1;73(17):5591–602. 23832665 10.1158/0008-5472.CAN-13-1351 PMC3766468

[35] Kim YH, Kim HK, Kim HY, Gawk H, Bae SH, Sim HW, FAK-Copy-Gain Is a Predictive Marker for Sensitivity to FAK Inhibition in Breast Cancer. Cancers. 2019 Sep 2;11(9):1288. 31480645 10.3390/cancers11091288 PMC6769494

[36] Kaveh F, Baumbusch LO, Nebdal D, Børresen-Dale AL, Lingjærde OC, Edvardsen H, A systematic comparison of copy number alterations in four types of female cancer. BMC Cancer. 2016 Nov 22;16:913. 27876019 10.1186/s12885-016-2899-4 PMC5120489

[37] Golubovskaya VM. Targeting FAK in human cancer: from finding to first clinical trials. Front Biosci Landmark Ed. 2014 Jan 1;19:687–706. 24389213 10.2741/4236 PMC3952878

[38] Agochiya M, Brunton VG, Owens DW, Parkinson EK, Paraskeva C, Keith WN, Increased dosage and amplification of the focal adhesion kinase gene in human cancer cells. Oncogene. 1999 Oct;18(41):5646–53. 10523844 10.1038/sj.onc.1202957

[39] Soria JC, Gan HK, Blagden SP, Plummer R, Arkenau HT, Ranson M, A phase I, pharmacokinetic and pharmacodynamic study of GSK2256098, a focal adhesion kinase inhibitor, in patients with advanced solid tumors. Ann Oncol. 2016 Dec 1;27(12):2268–74. 27733373 10.1093/annonc/mdw427

[40] Shimizu T, Fukuoka K, Takeda M, Iwasa T, Yoshida T, Horobin J, A first-in-Asian phase 1 study to evaluate safety, pharmacokinetics and clinical activity of VS-6063, a focal adhesion kinase (FAK) inhibitor in Japanese patients with advanced solid tumors. Cancer Chemother Pharmacol. 2016;77:997–1003. 27025608 10.1007/s00280-016-3010-1 PMC4844649

[41] Gerber DE, Camidge DR, Morgensztern D, Cetnar J, Kelly RJ, Ramalingam SS, Phase 2 Study of the Focal Adhesion Kinase Inhibitor Defactinib (VS-6063) in Previously Treated Advanced KRAS Mutant Non-small Cell Lung Cancer. Lung Cancer Amst Neth. 2020 Jan;139:60–7. 10.1016/j.lungcan.2019.10.033 PMC6942685 31739184

[42] Chapman NM, Houtman JCD. Functions of the FAK family kinases in T cells: beyond actin cytoskeletal rearrangement. Immunol Res. 2014 Aug;59(1–3):23–34. 24816556 10.1007/s12026-014-8527-y PMC4125427

[43] Chapman NM, Connolly SF, Reinl EL, Houtman JCD. Focal adhesion kinase negatively regulates Lck function downstream of the T cell antigen receptor. J Immunol Baltim Md 1950. 2013 Dec 15;191(12):10.4049/jimmunol.1301587. PMC3865716 24227778

[44] Gett AV, Sallusto F, Lanzavecchia A, Geginat J. T cell fitness determined by signal strength. Nat Immunol. 2003 Apr;4(4):355–60. 12640450 10.1038/ni908

[45] Ozmadenci D, Shankara Narayanan JS, Andrew J, Ojalill M, Barrie AM, Jiang S, Tumor FAK orchestrates immunosuppression in ovarian cancer via the CD155/TIGIT axis. Proc Natl Acad Sci. 2022 Apr 26;119(17):e2117065119. 35467979 10.1073/pnas.2117065119 PMC9169934

[46] Mohan N, Hosain S, Zhao J, Shen Y, Luo X, Jiang J, Atezolizumab potentiates Tcell-mediated cytotoxicity and coordinates with FAK to suppress cell invasion and motility in PD-L1+ triple negative breast cancer cells. OncoImmunology. 2019 Sep 2;8(9):e1624128. 31428520 10.1080/2162402X.2019.1624128 PMC6685513

[47] Wei Y, Wang Y, Liu N, Qi R, Xu Y, Li K, A FAK Inhibitor Boosts Anti-PD1 Immunotherapy in a Hepatocellular Carcinoma Mouse Model. Front Pharmacol. 2022 Jan 18;12:820446. 35115949 10.3389/fphar.2021.820446 PMC8804348

[48] Serrels B, McGivern N, Canel M, Byron A, Johnson SC, McSorley HJ, IL-33 and ST2 mediate FAK-dependent antitumor immune evasion through transcriptional networks. Sci Signal. 2017 Dec 5;10(508):eaan8355. 29208683 10.1126/scisignal.aan8355 PMC6128400

[49] Serrels A, Lund T, Serrels B, Byron A, McPherson RC, von Kriegsheim A, Nuclear FAK Controls Chemokine Transcription, Tregs, and Evasion of Anti-tumor Immunity. Cell. 2015 Sep;163(1):160–73. 26406376 10.1016/j.cell.2015.09.001 PMC4597190

[50] Canel M, Sławińska AD, Lonergan DW, Kallor AA, Upstill-Goddard R, Davidson C, FAK suppresses antigen processing and presentation to promote immune evasion in pancreatic cancer. Gut. 2024 Jan;73(1):131–55. 10.1136/gutjnl-2022-327927 PMC10715489 36977556

[51] Bagaev A, Kotlov N, Nomie K, Svekolkin V, Gafurov A, Isaeva O, Conserved pan-cancer microenvironment subtypes predict response to immunotherapy. Cancer Cell. 2021 Jun 14;39(6):845–65.e7. 34019806 10.1016/j.ccell.2021.04.014

[52] Park JS, Kim IK, Han S, Park I, Kim C, Bae J, Normalization of Tumor Vessels by Tie2 Activation and Ang2 Inhibition Enhances Drug Delivery and Produces a Favorable Tumor Microenvironment. Cancer Cell. 2016 Dec 12;30(6):953–67. 27960088 10.1016/j.ccell.2016.10.018

[53] Li AQ, Fang JH. Anti-angiogenic therapy enhances cancer immunotherapy: Mechanism and clinical application. Interdiscip Med. 2024;2(1):e20230025.

[54] Lee WS, Yang H, Chon HJ, Kim C. Combination of anti-angiogenic therapy and immune checkpoint blockade normalizes vascular-immune crosstalk to potentiate cancer immunity. Exp Mol Med. 2020 Sep;52(9):1475–85. 32913278 10.1038/s12276-020-00500-y PMC8080646

[55] Lim ST, Chen XL, Tomar A, Miller NLG, Yoo J, Schlaepfer DD. Knock-in Mutation Reveals an Essential Role for Focal Adhesion Kinase Activity in Blood Vessel Morphogenesis and Cell Motility-Polarity but Not Cell Proliferation. J Biol Chem. 2010 Jul 9;285(28):21526–36. 20442405 10.1074/jbc.M110.129999 PMC2898428

[56] Zhao X, Peng X, Sun S, Park AYJ, Guan JL. Role of kinase-independent and -dependent functions of FAK in endothelial cell survival and barrier function during embryonic development. J Cell Biol. 2010 Jun 14;189(6):955–65. 20530207 10.1083/jcb.200912094 PMC2886345

[57] Shen TL, Park AYJ, Alcaraz A, Peng X, Jang I, Koni P, Conditional knockout of focal adhesion kinase in endothelial cells reveals its role in angiogenesis and vascular development in late embryogenesis. J Cell Biol. 2005 Jun 20;169(6):941–52. 15967814 10.1083/jcb.200411155 PMC2171636

[58] Sun S, Wu HJ, Guan JL. Nuclear FAK and its kinase activity regulate VEGFR2 transcription in angiogenesis of adult mice. Sci Rep. 2018 Feb 7;8:2550. 29416084 10.1038/s41598-018-20930-z PMC5803223

[59] Tavora B, Batista S, Reynolds LE, Jadeja S, Robinson S, Kostourou V, Endothelial FAK is required for tumour angiogenesis. EMBO Mol Med. 2010 Dec;2(12):516–28. 21154724 10.1002/emmm.201000106 PMC3377344

[60] Lu C, Bonome T, Li Y, Kamat AA, Han LY, Schmandt R, Gene Alterations Identified by Expression Profiling in Tumor-Associated Endothelial Cells from Invasive Ovarian Carcinoma. Cancer Res. 2007 Feb 16;67(4):1757–68. 17308118 10.1158/0008-5472.CAN-06-3700

[61] Jean C, Chen XL, Nam JO, Tancioni I, Uryu S, Lawson C, Inhibition of endothelial FAK activity prevents tumor metastasis by enhancing barrier function. J Cell Biol. 2014 Jan 20;204(2):247–63. 24446483 10.1083/jcb.201307067 PMC3897185

[62] Pedrosa AR, Bodrug N, Gomez-Escudero J, Carter EP, Reynolds LE, Georgiou PN, Tumor Angiogenesis Is Differentially Regulated by Phosphorylation of Endothelial Cell Focal Adhesion Kinase Tyrosines-397 and −861. Cancer Res. 2019 Sep 3;79(17):4371–86. 31189647 10.1158/0008-5472.CAN-18-3934

[63] Hoeben A, Landuyt B, Highley MS, Wildiers H, Oosterom ATV, Bruijn EAD. Vascular Endothelial Growth Factor and Angiogenesis. Pharmacol Rev. 2004 Dec 1;56(4):549–80. 15602010 10.1124/pr.56.4.3

[64] Chen XL, Nam JO, Jean C, Lawson C, Walsh CT, Goka E, VEGF-induced vascular permeability is mediated by FAK. Dev Cell. 2012 Jan 17;22(1):146–57. 22264731 10.1016/j.devcel.2011.11.002 PMC3266538

[65] Cabrita MA, Jones LM, Quizi JL, Sabourin LA, McKay BC, Addison CL. Focal adhesion kinase inhibitors are potent anti-angiogenic agents. Mol Oncol. 2011 Dec;5(6):517–26. 22075057 10.1016/j.molonc.2011.10.004 PMC5528320

[66] Halder J, Lin YG, Merritt WM, Spannuth WA, Nick AM, Honda T, Therapeutic Efficacy of a Novel Focal Adhesion Kinase Inhibitor TAE226 in Ovarian Carcinoma. Cancer Res. 2007 Nov 15;67(22):10976–83. 18006843 10.1158/0008-5472.CAN-07-2667

[67] Henke E, Nandigama R, Ergün S. Extracellular Matrix in the Tumor Microenvironment and Its Impact on Cancer Therapy. Front Mol Biosci. 2020 Jan 31;6:160. 32118030 10.3389/fmolb.2019.00160 PMC7025524

[68] Lagares D, Busnadiego O, García-Fernández RA, Kapoor M, Liu S, Carter DE, Inhibition of focal adhesion kinase prevents experimental lung fibrosis and myofibroblast formation. Arthritis Rheum. 2012 May;64(5):1653–64. 22492165 10.1002/art.33482 PMC3338902

[69] Stokes JB, Adair SJ, Slack-Davis JK, Walters DM, Tilghman RW, Hershey ED, Inhibition of Focal Adhesion Kinase by PF-562,271 Inhibits the Growth and Metastasis of Pancreatic Cancer Concomitant with Altering the Tumor Microenvironment. Mol Cancer Ther. 2011 Nov;10(11):2135–45. 21903606 10.1158/1535-7163.MCT-11-0261 PMC3213273

[70] Qiao M, Zhou F, Liu X, Jiang T, Wang H, Li X, Targeting focal adhesion kinase boosts immune response in KRAS/LKB1 co-mutated lung adenocarcinoma via remodeling the tumor microenvironment. Exp Hematol Oncol. 2024 Jan 30;13:11. 38291516 10.1186/s40164-023-00471-6 PMC10826079

[71] Wang X, Li N, Liu YH, Wu J, Liu QG, Niu JB, Targeting focal adhesion kinase (FAK) in cancer therapy: A recent update on inhibitors and PROTAC degraders. Eur J Med Chem. 2024 Oct 5;276:116678. 39029337 10.1016/j.ejmech.2024.116678

[72] Yang M, Xiang H, Luo G. Targeting focal adhesion kinase (FAK) for cancer therapy: FAK inhibitors, FAK-based dual-target inhibitors and PROTAC degraders. Biochem Pharmacol. 2024 Jun 1;224:116246. 38685282 10.1016/j.bcp.2024.116246

[73] Banerjee S, Krebs MG, Greystoke A, Garces AI, Perez VS, Terbuch A, Defactinib with avutometinib in patients with solid tumors: the phase 1 FRAME trial. Nat Med. 2025 Jun 27;1–7. 40579546 10.1038/s41591-025-03763-y PMC12443583

[74] Lubrano S, Cervantes-Villagrana RD, Faraji F, Ramirez S, Sato K, Adame-Garcia SR, FAK inhibition combined with the RAF-MEK clamp avutometinib overcomes resistance to targeted and immune therapies in BRAF V600E melanoma. Cancer Cell. 2025 Mar 10;43(3):428–45.e6. 40020669 10.1016/j.ccell.2025.02.001 PMC11903146

[75] Cheng B, Lv J, Xiao Y, Song C, Chen J, Shao C. Small molecule inhibitors targeting PD-L1, CTLA4, VISTA, TIM-3, and LAG3 for cancer immunotherapy (2020–2024). Eur J Med Chem. 2025 Feb 5;283:117141. 39653621 10.1016/j.ejmech.2024.117141

[76] Lechertier T, Reynolds LE, Kim H, Pedrosa AR, Gómez-Escudero J, Muñoz-Félix JM, Pericyte FAK negatively regulates Gas6/Axl signalling to suppress tumour angiogenesis and tumour growth. Nat Commun. 2020 Jun 4;11(1):2810. 32499572 10.1038/s41467-020-16618-6 PMC7272651

[77] Kato T, Sato T, Yokoi K, Sekido Y. E-cadherin expression is correlated with focal adhesion kinase inhibitor resistance in Merlin-negative malignant mesothelioma cells. Oncogene. 2017 Sep;36(39):5522–31. 28553954 10.1038/onc.2017.147

